# Pseudoaneurysm of the profunda femoris artery following direct anterior approach total hip arthroplasty: a report of two cases

**DOI:** 10.1093/jscr/rjaf942

**Published:** 2026-04-15

**Authors:** Qiu Huang, Xiaoyu Li, Yongcai Wang, Wenhui Zhu

**Affiliations:** Department of Joint Surgery, The People’s Hospital of Leshan, 639 Huian Road, Shizhong District, Leshan, Sichuan 614000, PR China; Department of Medical Insurance, The People’s Hospital of Leshan, 639 Huian Road, Shizhong District, Leshan, Sichuan 614000, PR China; Department of Joint Surgery, The People’s Hospital of Leshan, 639 Huian Road, Shizhong District, Leshan, Sichuan 614000, PR China; Department of Sports Medicine, Huashan Hospital, Fudan University, 12 Urumqi Middle Road, Jingan District, Shanghai 200040, PR China

**Keywords:** pseudoaneurysm, profunda femoris artery, direct anterior approach, total hip arthroplasty

## Abstract

Pseudoaneurysm of the profunda femoris artery (PFA) is a rare but potentially serious complication of total hip arthroplasty (THA). Due to the atypical symptoms, diagnosis and treatment are often delayed. The direct anterior approach (DAA) for THA has grown increasingly popular, largely owing to its minimally invasive advantages. Here, we present two cases of pseudoaneurysm involving perforating branches of the PFA following DAA-THA. The first case underwent a tortuous diagnostic process, ultimately resolved by digital subtraction angiography (DSA)-guided open vascular ligation. In contrast, the second case was successfully treated with endovascular embolization immediately after DSA confirmation.

## Introduction

Total hip arthroplasty (THA) significantly improves quality of life for patients with end-stage hip disease. The direct anterior approach (DAA), favored for its muscle-sparing technique and rapid recovery, has gained widespread adoption. However, compared to traditional approaches, DAA requires aggressive soft tissue retraction to expose the hip joint, potentially increasing the risk of mechanical injury to adjacent vasculature, including the PFA and its perforating branches [1]. This report presents two cases of PFA branch pseudoaneurysms following DAA-THA in the lateral position, emphasizing diagnostic challenges and therapeutic approaches.

## Case presentation

### Case 1

**Figure 1:**
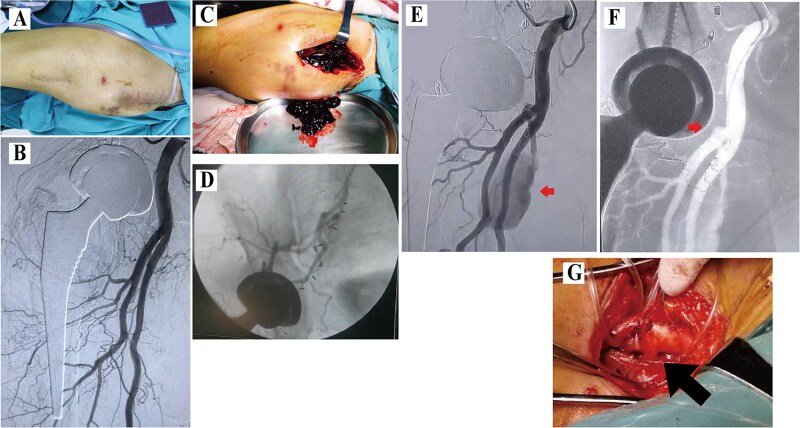
Data of Case 1. (A) Swelling at the proximal thigh; (B)initial DSA showing no pseudoaneurysm; (C) intraoperative hematoma; (D) intraoperative angiography and obturator artery branch embolization using C-arm fluoroscopy; (E) second DSA demonstrating a pseudoaneurysm; (F) failed coil embolization due to tortuosity; (G) surgical ligation.

A 58-year-old male patient with a body mass index (BMI) of 28.45 kg/m2 underwent right DAA-THA for femoral head avascular necrosis. The procedure was uneventful, with normal postoperative imaging and neurovascular exams. On postoperative day 14, he suddenly developed severe right groin pain and rapid thigh swelling. Ultrasound revealed a hematoma without evidence of pseudoaneurysm. Conservative management (analgesia, hemostatic agents, discontinuation of rivaroxaban, and immobilization) resulted in partial improvement. However, on postoperative day 21, symptoms recurred with worsened swelling and pain (Fig. 1A). Digital subtraction angiography (DSA) showed no abnormalities (Fig. 1B). Surgical exploration was subsequently performed, revealing a large hematoma accompanied by refractory deep tissue oozing of blood (Fig. 1C). Intraoperative angiography using a C-arm also demonstrated no contrast extravasation or pseudoaneurysm formation. Hemostasis was achieved with C-arm-guided obturator artery branch embolization combined with compression (Fig. 1D). On postoperative day 37, the patient developed recurrent groin pain and proximal thigh swelling. Emergency DSA identified a PFA pseudoaneurysm (Fig. 1E), and an attempt at endovascular coil embolization failed due to tortuous anatomy (Fig. 1F). Open surgery via an inguinal incision to ligate the culprit vessel successfully controlled hemorrhage (Fig. 1G). Symptoms resolved completely at 3-month follow-up.

### Case 2

**Figure 2:**
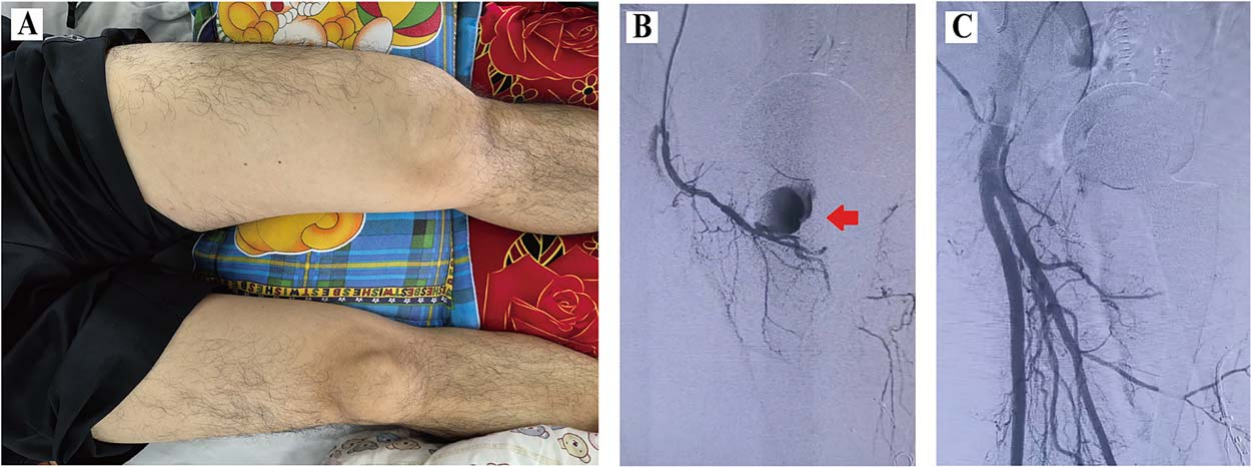
Data of Case 2. (A) Swelling of the left thigh; (B) DSA confirming pseudoaneurysm; (C) post-embolization image.

A 59-year-old male patient with a BMI of 27.89 kg/m^2^ underwent left DAA-THA for femoral head avascular necrosis. On postoperative day 18, the patient experienced sudden left groin pain and thigh swelling during weight-bearing (Fig. 2A). Emergency DSA confirmed a pseudoaneurysm of the perforating branch of the PFA (Fig. 2B). Superselective microcatheterization of the distal perforating branch was performed, followed by successful coil embolization proximal and distal to the pseudoaneurysm neck (Fig. 2C). Complete resolution was observed at the 3-month follow-up.

## Discussion

Iatrogenic injury is a leading cause of PFA pseudoaneurysms [1]. In both cases reported here, pseudoaneurysms occurred after DAA-THA performed in the lateral position. The underlying mechanisms may include: (i) Anatomical vulnerability and mechanical strain: During DAA-THA in the lateral-position, exposure of the femoral component requires extreme adduction, extension, and external rotation of the limb, which may generate aggressive traction forces on the posteromedial soft tissues and then leading to tears of the PFA or its perforating branches. (ii) Intraoperative retractor-related trauma: To achieve surgical field exposure multiple acetabular retractors are placed around the acetabulum, which may directly compress or lacerate the PFA.

Due to the deep anatomical location and rupture at presentation, neither case exhibited classic pseudoaneurysm signs such as a pulsatile mass [2]. Instead, patients presented with acute-onset groin pain and proximal thigh swelling, clinically overlapping with postoperative hematoma. In Case 1, pre-operative ultrasound and DSA failed to detect the pseudoaneurysm. Intraoperative C-arm angiography during open exploratory surgery also showed no abnormalities. Potential reasons include: temporary hemostasis due to elevated local pressure from the hematoma, complete pseudoaneurysm rupture. Subsequent DSA clearly identified the pseudoaneurysm and its feeding vessel. Although endovascular coil embolization was attempted, tortuous vessel anatomy precluded successful catheterization, necessitating open surgical ligation. In contrast, Case 2 underwent immediate DSA upon symptom onset, enabling prompt embolization. In summary, DSA-guided embolization is the preferred diagnostic and therapeutic approach for PFA pseudoaneurysms, offering precision, minimal invasiveness, and reduced infection risk [3]. Open surgical exploration is generally discouraged as the initial approach. However, for institutions lacking endovascular capabilities or in cases with complex vascular anatomy, CT or DSA-guided identification of the culprit vessel followed by targeted open ligation remains a viable alternative [1, 3, 4].
